# Optimizing implant positioning in total hip arthroplasty via the direct anterior approach: The role and technique of conventional traction table and fluoroscopy

**DOI:** 10.1186/s42836-024-00293-9

**Published:** 2025-02-07

**Authors:** Seiya Ishii, Tomonori Baba, Koju Hayashi, Yasuhiro Homma, Osamu Muto, Muneaki Ishijima

**Affiliations:** 1 Department of Orthopaedic Surgery, Yokohama Tsurugamine Hospital, Kanagawa, 241-0011 Japan; 2 Department of Orthopaedics, Faculty of Medicine, Juntendo University, Tokyo, 113-0033 Japan; 3 Department of Medicine for Orthopaedics and Motor Organ, Juntendo University Graduate School of Medicine, Tokyo, 113-0033 Japan

**Keywords:** Direct anterior approach, Total hip arthroplasty, Fluoroscopy, Component positioning, Component safe zone

## Abstract

**Background:**

Precise implant positioning during total hip arthroplasty (THA) is an important factor influencing dislocation rate and long-term implant survival. Although a special carbon fiber traction table for THA improves the accuracy of implant positioning, it is too expensive. We aimed to report the accuracy of cup positioning and complication rate in patients undergoing THA via the direct anterior approach using a conventional noncarbon fiber traction table, which is generally used for osteosynthesis of femoral fractures.

**Methods:**

This retrospective study included 101 patients who received primary THA via the direct anterior approach using a conventional traction table with fluoroscopy between July 2022 and October 2024. Two observers evaluated radiological outcomes using postoperative anteroposterior X-rays. The intraclass correlation coefficients of cup positioning angles were calculated (inclination: 0.92, anteversion: 0.89 for intra-observer agreement; inclination: 0.91, anteversion: 0.85 for inter-observer agreement). Complications were defined as dislocation, periprosthetic fracture, ankle fracture, implant loosening, nerve injury, surgical site infection, deep vein thrombosis, and revision surgery for any reason.

**Results:**

Radiographic analysis showed an average cup inclination of 38.1° ± 4.1° (99.0% within Lewinnek’s safe zone). The average cup anteversion was 12.0° ± 4.7° (97.0% within Lewinnek’s safe zone). None of the patients experienced any complications.

**Conclusion:**

The use of a conventional traction table to perform THA using fluoroscopy may not interfere with precise cup positioning. This technique, which does not require a special carbon fiber traction table for THA, could be a feasible alternative for performing THA at general hospitals.

## Introduction

Total hip arthroplasty (THA) represents one of the most successful surgical interventions in the twenty-first century, effectively improving the quality of life of patients with end-stage hip disorders [1]. Because of improved surgical techniques and implant materials, favorable mid- to long-term clinical outcomes have been achieved following THA, with an implant survival rate of > 90% at 15 years [2]. Precise implant positioning and restoration of hip biomechanics during THA have been identified as two important factors that influencing the dislocation rate, abductor function, polyethylene wear, implant impingement, and long-term implant survival [3–6]. During conventional THA, an intraoperative alignment guide and anatomical bony landmarks were used to assess the implant positioning. However, the reproducibility of implant placement using this method remains low because it highly depends on surgeon’s experience and intraoperative tilting of the pelvis [7]. Callanan et al. reported that only 49% (917/1883 hips) of acetabular implants were inserted inside of the “Lewinnek’s safe zone”, which is the desired angle range [8]. Various techniques to ensure accurate implant placement have been reported, one of which involves the use of a carbon fiber traction table specialized for direct anterior approach (DAA)-THA in combination with fluoroscopy, which assists in achieving accurate implant positioning [9–15]. Moreover, robotic surgery has recently been introduced at several facilities to improve the accuracy of implant positioning [16–19]. However, THA using a carbon fiber traction table for DAA-THA is costly, making it difficult to introduce the table into general community hospitals. Therefore, we performed DAA-THA using a conventional non-carbon traction table, which has been used in most hospitals for osteosynthesis of femoral fractures. No previous study has examined the clinical results of THA using this conventional traction table. The current study aimed to evaluate the accuracy of implant positioning and the safety of DAA-THA using a conventional traction table with fluoroscopy.

## Material and methods

After approval by the institutional review board, a retrospective cohort study was conducted. We reviewed 194 consecutive primary THA procedures performed at our hospital between July 2022 and October 2024. Although a carbon fiber traction table has been used in cases of DAA-THA for the initial series, we used a conventional non-carbon fiber traction table, considering the high cost of the former. Patients who received THA via the posterior approach (*n* = 86), DAA-THA using a carbon fiber traction table (*n* = 6), or DAA-THA using a curved short stem (*n* = 1) were excluded. All the remaining patients received DAA-THA performed by a single surgeon (S.I.) and were included in this study.

DAA was performed as previously reported using the distal portion of the Smith–Petersen approach [20]. The patient was positioned supine on a standard operating table [21], and the patient’s foot was secured to the boot of a conventional non-carbon fiber traction table designed for osteosynthesis of femoral fractures.

**Fig. 1:**
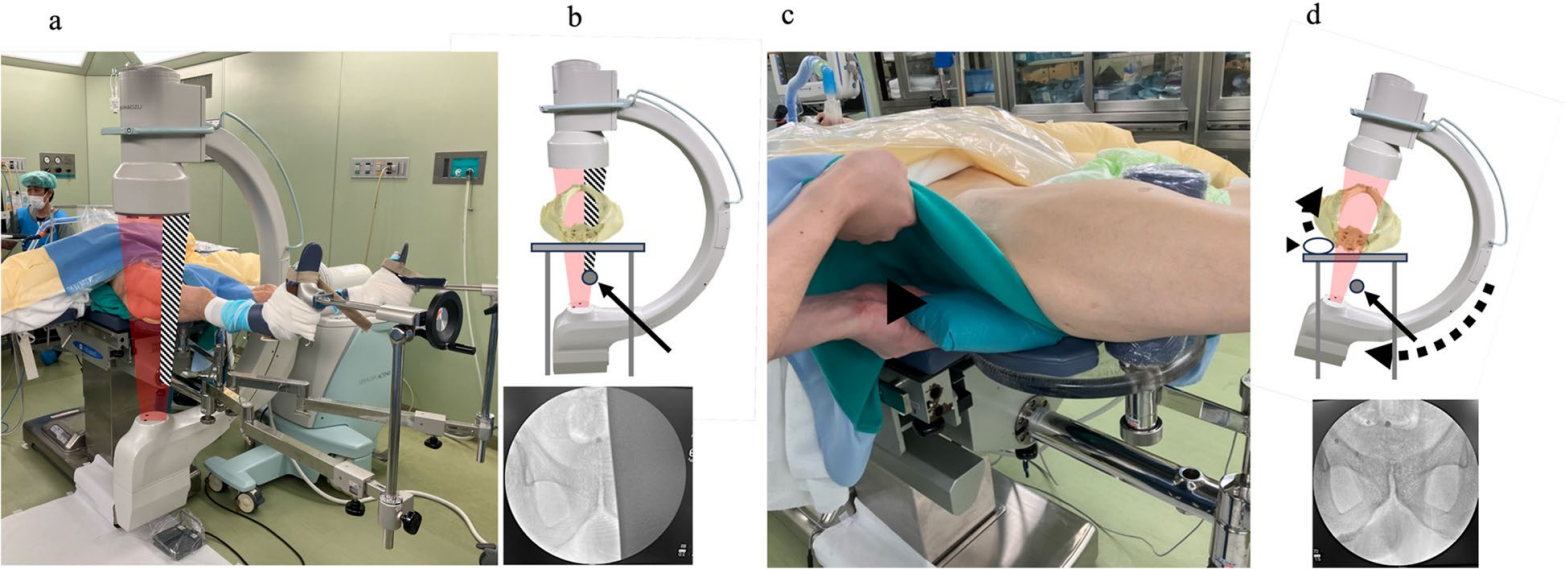
Position and fluoroscopic settings. **a**, **b** Obstruction of fluoroscopy radiation by a metal rod under the pelvis. **c**, **d** The pelvis was tilted by inserting a pillow under the affected hip, which allowed us to display the bilateral obturator foramina on the monitor. Black arrow: a metal rod of the traction table. Black arrowhead: a pillow inserted under the hip

In the traction table specialized for DAA, carbon fiber was used as the rod to support the lower extremity owing to its high rigidity and radiolucency. To maintain the rigidity of the conventional traction table, a radiopaque metal was used instead of carbon fiber. Therefore, in some cases, both obturator foramina were not visible using fluoroscopy with the conventional traction table. To address this, the hip of the affected side was elevated by placing a pillow under it and the C-arm was rotated accordingly to display the whole obturator foramina on the monitor (Fig. 1).

**Fig. 2:**
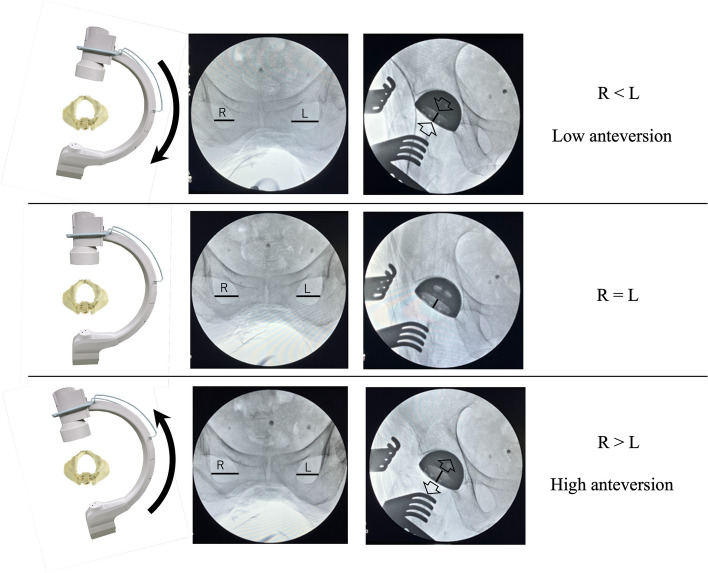
Changes in component appearance due to the angle of incidence of the radiation. R: Width of the obturator foramina on the right side. L: Width of the obturator foramina on the left side

Recreation of neutral rotation of the pelvis and preoperative patient-specific sagittal pelvic tilt against the C-arm during the surgical procedure enhances accurate component positioning [22]. To achieve this, we adjusted the angle of the C-arm in the axial plane so that both obturator foramina appeared as mirror images on the C-arm monitor (Fig. 2). Sagittal rotation of the C-arm was also adjusted to ensure that the distance from the coccyx to the pubic symphysis matched that on the preoperative supine anteroposterior radiograph. This adjustment of the angle of the C-arm to the pelvis was performed before acetabular reaming and again before cup placement.

**Fig. 3:**
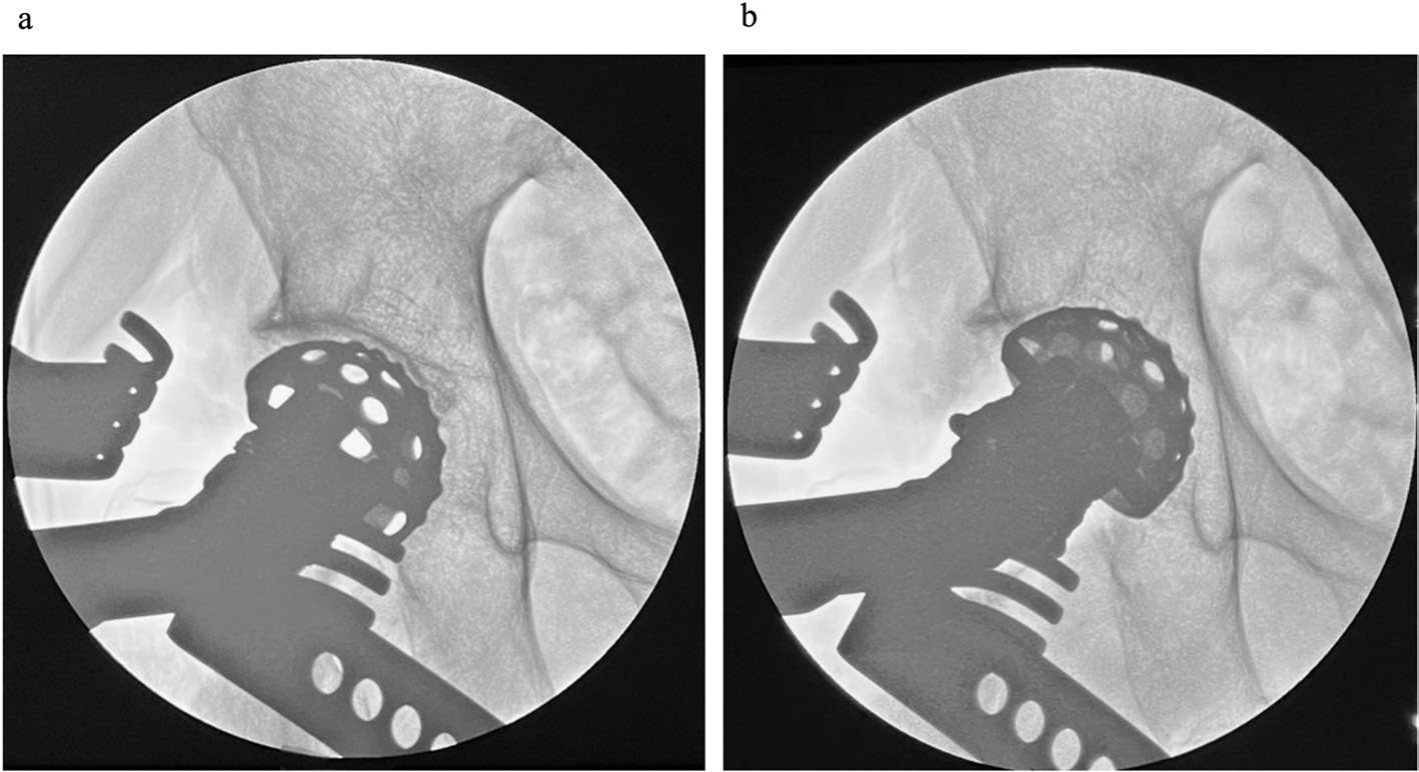
Acetabular reaming under fluoroscopic guidance. **a** Before reaming. **b** Optimal medialization of the reamer

After conforming to the joint line using fluoroscopy, a skin incision started at the level of the joint line and extended 8 cm distally. The sheath of the tensor fascia lata was incised longitudinally along the direction of the muscle fibers, and the lateral circumflex femoral artery was controlled with electrocautery. The joint capsule was incised in a Y-shaped manner, preserving the medial band of the iliofemoral ligament. Next, femoral neck osteotomy was performed after checking the appropriate level using fluoroscopy, and the femoral head was removed anteriorly. After resecting the labrum, acetabular reaming and cup implantation were performed under fluoroscopic guidance. The acetabular reamer was carefully medialized into the acetabulum under fluoroscopic guidance while confirming adequate initial fixation and not penetrating the medial acetabular wall (Fig. 3). After the acetabular reaming was performed, a cup was inserted under fluoroscopic control. For patients aged > 65 years, a dual-mobility system was used (DM; Dual-mobility system with E1 Active Articulation bearing, Zimmer-Biomet, Warsaw, IN, USA). In cases of DM, a metal liner was placed under fluoroscopic guidance.

To prepare for stem insertion, the leg was gently retracted in external rotation at 45°. The calcar femorale was elevated using a single hook retractor, and another retractor was then inserted through a 2-cm incision in the superior capsule into the region between the insertion point of the tendons of gluteus medius and piriformis, known as the “bald spot” [23]. The traction of the leg was then completely released, and the foot was further externally rotated. If this external rotation was not sufficient to adequately expose the femur, soft tissue release from the femur was performed as previously reported [24]. After confirming that the leg was not retracted, it descendedso that the hip joint could be extended to 30°. We then inserted a rasp that was one size smaller than that planned preoperatively. A trial head and neck were assembled, and trial reduction was performed. During this process, we fluoroscopically assessed the stem size, alignment, depth of insertion, and any leg length discrepancies. Anterior stability was assessed by externally rotating the foot at 90° with 10° hip extension. After the stem and head were assembled, the surgical wound was irrigated with a diluted 0.35% povidone-iodine solution for 3 min, followed by irrigation with normal saline. The anterior capsule was repaired in all cases. Moreover, to prevent postoperative dislocation, it was repaired tightly, particularly in cases of intraoperative instability.

Uncemented cups (G7 acetabular components, Zimmer-Biomet, Warsaw, IN, USA) and uncemented or cemented stems (85 cases–Avenir complete hip system, Zimmer-Biomet, Warsaw, IN, USA; 16 cases–CMK Modular stem, Zimmer-Biomet, Warsaw, IN, USA) were used in the study patients. Except for a single case with an intraoperative fracture, all patients were allowed to be fully weight-bearing immediately after surgery.

Radiographic outcomes were assessed using postoperative anteroposterior and Lauenstein radiographs obtained in the supine position. We radiographically evaluated inclination and anteversion of the acetabular component, stem alignment, and leg length discrepancy. The primary outcome of this study was the accuracy of cup positioning achieved after THA. Radiographic cup anteversion was calculated as described by Liaw et al. [25]. The position of the cup was assessed, based on the “safe zone” defined by Lewinnek et al., as an inclination of 40° ± 10° and an anteversion of 15° ± 10° [3]. Stem alignment was assessed by measuring the angle between the long axis of the stem and the long axis of the femur. All measurements were conducted using the hospital’s computerized picture archiving and communication system (SYNAPSE; Fujifilm, Tokyo, Japan) by two authors (S.I. and K.H.). The intraclass correlation coefficient (ICC) was used to measure inter- and intra-observer reliability. The measurements had a “good” to “excellent” ICC for intra-observer (Inclination; 0.92, Anteversion; 0.89) and inter-observer (Inclination; 0.91, Anteversion; 0.85) correlation [26].

Demographic data of the included patients (age, height, weight, body mass index, sex, etiology, and operative side) and surgical and radiographic data were collected from the medical records. Surgical outcomes included surgical time, intraoperative blood loss, estimated total blood loss, duration of intraoperative fluoroscopic use, and complications, such as dislocation, intraoperative and postoperative periprosthetic fracture, ankle fracture, implant loosening, pudendal nerve injury, femoral nerve injury, sciatic nerve injury, surgical site infection, deep vein thrombosis, and revision surgery for any reason.

JMP Pro 18 for Macintosh was used for data analysis. Continuous data except blood loss were presented as the mean and standard deviation (SD) along with the range, and categorical variables were expressed as the absolute and relative frequency. Normality was assessed using the Shapiro–Wilk test. Because of non-normal distribution, blood loss was reported as the median and interquartile range (IQR).

## Results

A total of 101 patients who underwent DAA-THA using a conventional traction table with a C-arm were included in this study. Demographic data, surgical outcomes, and radiographic outcomes are summarized in Table 1. The average cup inclination was 38.1° ± 4.1° (range, 31°–51°), and 100/101 (99.0%) had an inclination between 30° and 50°. The average cup anteversion was 12.0° ± 4.7° (range, 1°–23°), and 98/101 (97.0%) had an anteversion between 5° and 25°. In total, 96.0% of the acetabular components were within the safe zone. One patient experienced an intraoperative acetabular fracture. In that case, the acetabular component was securely fixed with multiple screws, and weight-bearing was started four weeks postoperatively. There were no cases of dislocation, intraoperative femoral or ankle fracture, postoperative periprosthetic fracture, implant loosening, nerve injury (including pudendal nerve, femoral nerve, and sciatic nerve injury), surgical site infection, deep vein thrombosis, or revision surgery. There were no surgical complications related to use of the traction table (Table 1).

**Table 1:** Basic characteristics

Parameters	Values
Demographic data	
No. of hips, n	101
Age (year)	76.2 ± 11.1 (50–96)
Height (cm)	155.5 ± 8.5 (135–180)
Weight (kg)	55.8 ± 13.5 (35–94)
BMI (kg/m)	23.0 ± 4.6 (14–37)
Sex, *n* (%)	
Men	20 (19.8)
Women	81 (80.2)
Etiology, *n* (%)	
Osteoarthritis	50 (49.5)
Osteonecrosis	1 (1.0)
Proximal femoral fracture	50 (49.5)
Operative side, *n* (%)	
Right	45 (44.6)
Left	56 (55.4)

The values are given as the mean and SD (with the range in parentheses), or the number of patients (with the percentage in parentheses). *BMI* Body Mass Index

## Discussion

The current study demonstrated a high accuracy of acetabular implant positioning and low complication risk after DAA-THA using a conventional traction table. The rates at which acetabular implants were positioned within the safe zone in the current study (inclination; 99.0%, anteversion; 97.0%, both; 96.0%) were not inferior to those previously reported for DAA-THA using a carbon fiber traction table (inclination; 68.6–96.3%, anteversion; 18.6–93.0%, both; 42.9%) [9–15] or robotic-assisted THA (inclination; 96.0–100%, anteversion; 77.0–98.0%, both; 77.0–98.0%) [16–19] (Table 2). The study suggested that DAA-THA using a non-carbon traction table would be non-inferior to those employing a carbon traction one or a robot in terms of implant survival, complication rates, blood loss, and operative time (Table 3). DAA-THA using a conventional traction table and fluoroscopy, which we termed as “Safe Anterior Approach with Fracture table, or SAAF” could be a cost-effective and reliable technique (Table 4).

**Table 2:** Clinical and radiographic outcomes

Parameters	Values
Clinical outcomes	
Surgery time (minutes)	73.9 ± 20.7 (48–175)
Intraoperative blood loss (mL)	133 (81–209)
Complications	
Dislocation, *n*	0
Intraoperative fracture, *n*	1
Acetabular fracture, *n* (%)	1
Femoral fracture, *n* (%)	0
Ankle fracture, *n*	0
Postoperative fracture, *n*	0
Implant loosening, *n*	0
Nerve injury, *n*	0
SSI, *n*	0
DVT, *n*	0
Revision surgery, *n*	0
Radiographic outcomes	
Cup alignment	
Cup inclination (degrees)	38.1 ± 4.1 (31–51)
Within the safe zone, *n* (%)	100 (99.0)
Cup anteversion (degrees)	12.0 ± 4.7 (1–23)
Within the safe zone, *n* (%)	98 (97.0)
Both of inclination and anteversion within the safe zone, *n* (%)	97 (96.0)
Stem alignment	
Coronal plane (degrees)	0.5 ± 1.7 (−3–5)
Sagittal plane (degrees)	1.3 ± 1.9 (−5–6)
Coronal plane in neutral (−3≦ angle ≦3), *n* (%)	93 (92.1)
Sagittal plane in neutral (−3≦ angle ≦3), *n* (%)	87 (86.1)
Leg length discrepancy (mm)	1.3 ± 6.3 (−20–16)

The values are given as the median and the interquartile range, the mean and SD (with the range in parentheses), or the number of patients (with the percentage in parentheses). *SSI* Surgical Site Infection, *DVT* Deep Vein Thrombosis

**Table 3:** Summary of previous studies comparing the accuracy of cup positioning in THA

				Inclination				Anteversion				Inclination and anteversion
Articles	Support instrument	Approach	Number of hips (n)	Average inclination angle (degree)	SD (degree)	Definition of the target zone (degree)	Within the target zone (%)	Average anteversion angle (degree)	SD (degree)	Definition of the target zone (degree)	Within the target zone (%)	Both within the target zone (%)
This study	Conventional traction table	DAA	101	38.1	4.1	30–50	99.0	12.0	4.7	5–25	97.0	96.0
Matta [9]	Carbon fiber traction table	DAA	494	42	4	35–50	96	19.4	5.2	10–25	93	NA
Woolson [10]	Carbon fiber traction table	DAA	247	44	NA	30–50	79	NA	NA	NA	NA	NA
Hamilton [11]	Carbon fiber traction table	DAA	100	44.2	5.0	30–50	90	17.6	4.5	5–25	92	NA
Cheng [12]	Carbon fiber traction table	DAA	35	46.2	6.1	30–50	68.6	24.6	8.8	5–25	NA	42.9
Lin [13]	Carbon fiber traction table	DAA	108	NA	NA	30–50	96.3	22.7	NA	5–25	63.9	NA
Wernly [14]	Carbon fiber traction table	DAA	75	43.7	4.3	30–50	93.3	32	7	5–25	18.6	NA
Moslemi [15]	Carbon fiber traction table	DAA	137	40.4	7.1	30–50	84.7	15.6	11.8	5–25	61.3	NA
Domb [16]	Robot assisted	DAA, PA	66	40.9	3.2	30–50	100	18.4	3.7	5–25	97.0	97.0
Illgen [17]	Robot assisted	PA	100	NA	NA	30–50	100	NA	NA	5–25	77.0	77.0
Stewart [18]	Robot assisted	DAA	100	NA	NA	30–50	96.0	NA	NA	5–25	91.0	87.0
Foissey [19]	Robot assisted	DAA	50	40.5	3.4	30–50	100	23.4	3.5	10–30	98.0	98.0

The number of the author’s name refers to the reference number. *NA* Not applicable

**Table 4:** Summary of previous studies comparing the clinical outcomes in THA

Articles	Traction table	Number of hips (n)	Age (years)	BMI (kg/m^2^)	Implant survival rate (%)	Surgical complication rate (%)	Dislocation rate (%)	Blood loss (mL)	Surgical time (min)	Follow-up (months)
This study	Conventional traction table	101	76.2	23.0	100	1.0	0	133	73	9.1
Matta [9]	Carbon fiber traction table	494	64	NA	NA	3.4	0.6	350	75	NA
Woolson [10]	Carbon fiber traction table	247	67.7	28.4	97.2	15.8	0	858	164	8
Hamilton [11]	Carbon fiber traction table	100	61.1	29	98	3.0	2.0	NA	NA	NA
Cheng [12]	Carbon fiber traction table	35	61	28	97.1	11.4	2.9	NA	125	3
Lin [13]	Carbon fiber traction table	108	61.2	28.1	NA	NA	NA	NA	81	NA
Wernly [14]	Carbon fiber traction table	75	70	26	96.0	6.7	0	746	142	43
Moslemi [15]	Carbon fiber traction table	137	65	25.1	NA	3.7	1.5	NA	NA	NA
Domb [16]	Robot-assisted	66	59.0	29.2	95.5	6.0	1.5	NA	NA	> 60
Illgen [17]	Robot-assisted	100	62.4	29.2	NA	0	0	358	143	27.6
Stewart [18]	Robot-assisted	100	62.2	29.6	NA	NA	NA	NA	NA	NA
Foissey [19]	Robot-assisted	50	66.5	26.9	NA	4	0	NA	112	12

The number of the author’s name reflects the reference number. *BMI* Body Mass Index, *NA* Not Applicable

A previous study reported that the use of intraoperative fluoroscopy for THA on a carbon fiber traction table had a limited impact on enhancing the accuracy of acetabular implant positioning [27]. However, the current study showed further improvements in accuracy, which may be attributed to the use of our techniques.

First, a neutral pelvic image must be obtained before implant insertion because the non-optimal angle of incidence of fluoroscopic radiation to the pelvis can lead to measurement errors in radiographic inclination and anteversion [22] (Fig. 2).

The angle between the pelvis and the horizontal plane in the supine position was unstable during THA [28]. Moreover, as the cup is inserted with traction on the lower extremity, the traction force may affect the pelvic tilt. Therefore, an optimal pelvic image should be displayed on the monitor just before acetabular implant placement.

**Fig. 4:**
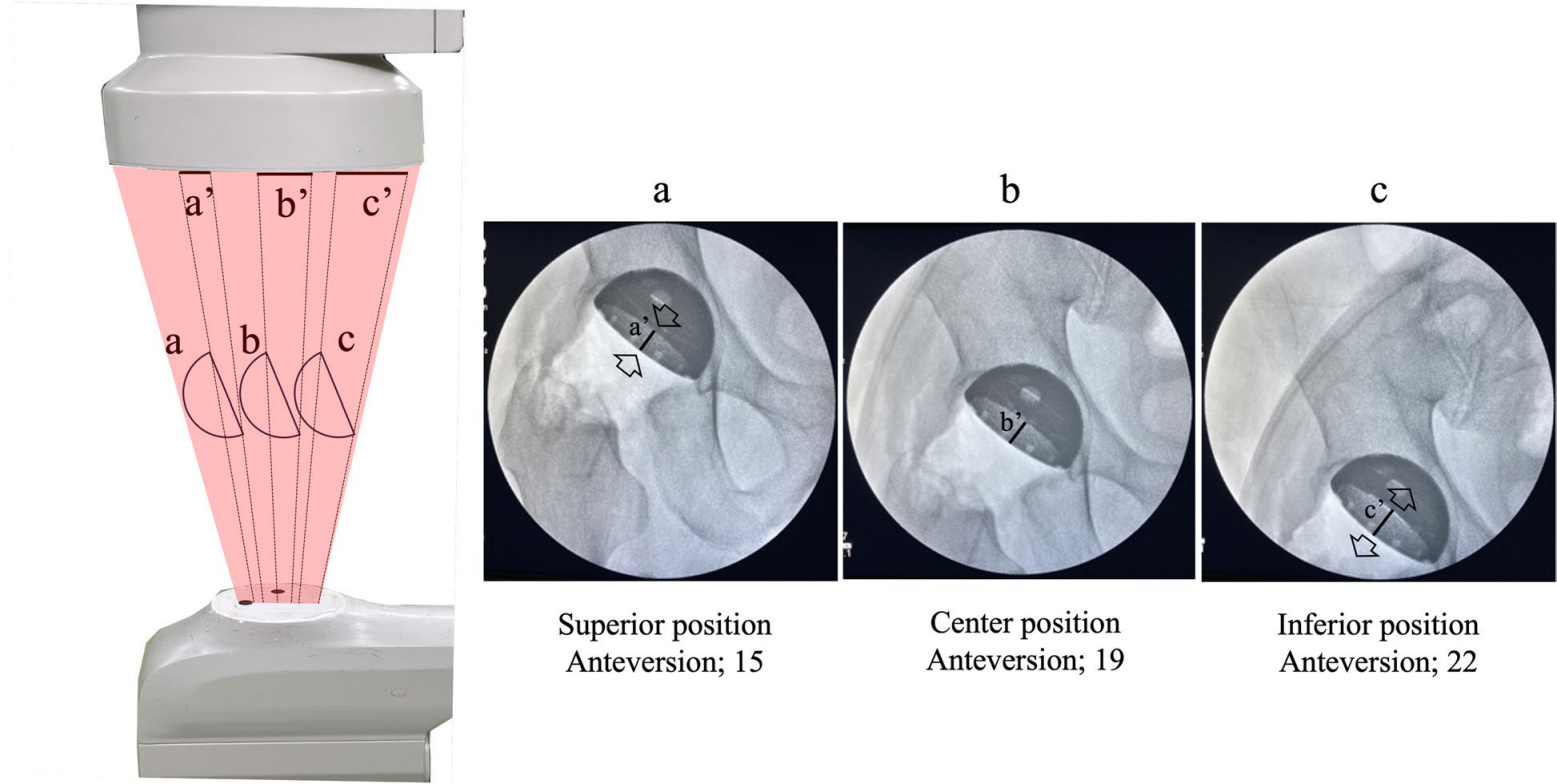
Different component appearances depend on the area on the monitor. **a** Superior position, low anteversion. **b** Center position. **c** Inferior position, high anteversion

Second, the object should always be at the center of the C-arm monitor. In an image intensifier system, X-rays emitted from a source pass through the human body and reach the center of the detector at a perpendicular angle. However, X-rays directed toward the edges deviate from this angle, resulting in differences in the radiographic inclination and anteversion of the cup displayed at the center compared to the periphery of the monitor (parallax error) (Fig. 4) [29]. Additionally, the peripheral area of the fluoroscopic monitor is distorted due to the curvature of the detector surface that receives X-rays (pin-cushion distortion) [30]. Therefore, the cup should be assessed at the center of the fluoroscopic monitor to improve the accuracy of the cup positioning.

## Limitations

This study has several limitations. First, all THA cases in this study were treated by a single surgeon and the sample size was small. Therefore, a cluster bias should be considered. Although the accuracy of cup positioning may depend on the surgeon’s surgical experience, most facilities should be able to reproduce this result by accurately following the techniques we described.

Second, the follow-up period was short (9.1 months). However, the length of the follow-up period did not influence the cup positioning angle, which was the primary outcome. Moreover, no intraoperative or immediate postoperative fractures were observed, and they were secondary outcomes. Future studies with a long follow-up period are warranted to determine long-term safety.

Third, using a traction table is associated with a risk of pudendal nerve injury resulting from direct compression by the peroneal post, leading to transient nerve palsy in the pubic region [31, 32]. However, no cases suffered from pudendal nerve injury in the present study. We believe that this was because the traction time was kept as short as possible. Dippmann et al. reported an average traction time of 98 min (range, 94 to 110), and 10% of their cases developed pudendal nerve injury [31]. Nicholson et al. studied the process of mechanical compression of a nerve and described that the duration of compression was an important risk factor for nerve dysfunction [32]. In this study, we only retracted the leg while inserting the posterior femoral retractor, inserting the acetabular reamer and component, and reducing dislocation after implant placement. Except for these traction procedures, the traction was released throughout the surgery. In most of our cases, the traction time lasted for < 15 min. THA using a traction table would have a low risk of pudendal nerve injury, provided that the traction time is short.

## Conclusion

Accurate implant positioning and low complication rates have been demonstrated in patients who underwent DAA-THA on a conventional traction table using fluoroscopy. This technique, which does not require an additional carbon fiber traction table or a robot, would be a feasible alternative for performing DAA-THA at general hospitals. Future studies to evaluate the effectiveness of DAA-THA on a conventional traction table in the comparative study should be performed.

## Data Availability

Data are available upon request from the corresponding author.
